# Structural characterization of self-assembled chain like Fe-FeOx Core shell nanostructure

**DOI:** 10.1186/s11671-019-3128-2

**Published:** 2019-09-09

**Authors:** Aiman Mukhtar, Xiao-Ming Cao, Tahir Mehmood, Da-shuang Wang, Kai-ming Wu

**Affiliations:** The State Key Laboratory of Refractories and Metallurgy, Hubei Province Key Laboratory of Systems Science in Metallurgical Process, International Research Institute for Steel Technology, Wuhan University of Science and Technology, Wuhan, People’s Republic of China

**Keywords:** 1-D nanostructures, Fe nanochains, Raman spectroscopy, Transmission electron microscopy

## Abstract

One of the big challenge of studying the core-shell iron nanostructures is to know the nature of oxide shell, i.e., whether it is γ-Fe_2_O_3_ (Maghemite), Fe_3_O_4_ (Magnetite), *α*-Fe_2_O_3_ (Hematite), or FeO (Wustite). By knowing the nature of iron oxide shell with zero valent iron core, one can determine the chemical or physical behavior of core-shell nanostructures. Fe core-shell nanochains (NCs) were prepared through the reduction of Fe^3+^ ions by sodium boro-hydride in aqueous solution at room atmosphere, and Fe NCs were further aged in water up to 240 min. XRD was used to study the structure of Fe NCs. Further analysis of core-shell nature of Fe NCs was done by TEM, results showed increase in thickness of oxide shell (from 2.5, 4, 6 to 10 nm) as water aging time increases (from 0 min, 120 min, 240 min to 360 min). The Raman spectroscopy was employed to study the oxide nature of Fe NCs. To further confirm the magnetite phase in Fe NCs, the Mössbauer spectroscopy was done on Fe NCs-0 and Fe NCs-6. Result shows the presence of magnetite in the sample before aging in water, and the sample after prolonged aging contains pure Hematite phase*.* It shows that prolonged water oxidation transforms the structure of shell of Fe NCs from mixture of Hematite and Magnetite in to pure hematite shell. The Magnetic properties of the Fe NCs were measured by VSM at 320 K. Because of high saturation magnetization (Ms) values, Fe NCs could be used as *r*_2_ contrasts agents for magnetic resonance imaging (MRI) in near future.

**Electronic supplementary material:**

The online version of this article (10.1186/s11671-019-3128-2) contains supplementary material, which is available to authorized users.

## Introduction

**Fig. 1:**
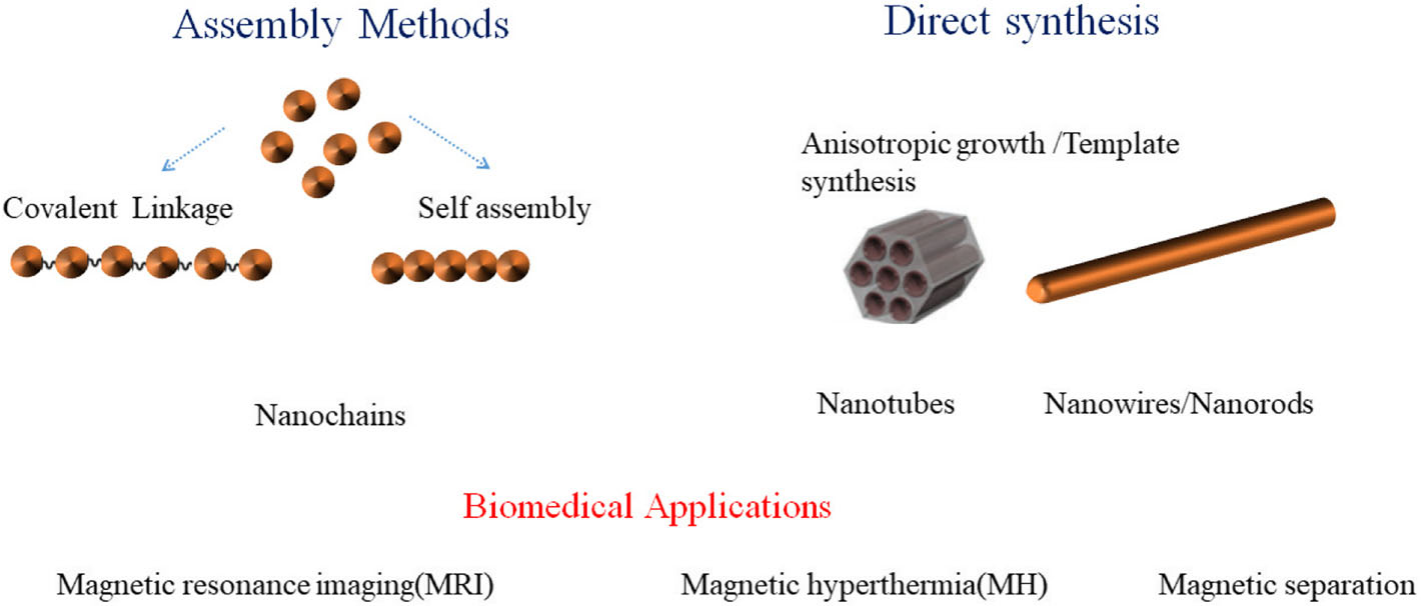
Highly shape anisotropic magnetic nanowires prepared through direct synthesis or assembly methods and their biomedical applications

Magnetic nanowires (NWs) either synthesized through template or assembly methods have large magnetic moments and shape anisotropy, as shown in Fig. 1. Previous work claim that chain-like structures with high aspect ratios are more efficient in biomedical applications especially for magnetic separation, magnetic hyperthermia (MH), and MRI than nanoparticles [1, 2]. For MH 1-D magnetic NWs can provide larger frictional reactive areas than zero-dimensional (0-D) nanoparticles. This permits one-dimensional (1-D) magnetic NWs to have a better heating efficiency, which reduces the treatment time of the cancer patients. Park et al. [3] reported that 1-D nanoworms show superior in vivo tumor-targeting ability than nanospheres with the similar diameter. Jeotikanta et al. prepared [4] Fe_3_O_4_ nanorods (NRs) by the hydrolysis of FeCl_3_ aqueous solution and functionalized with polyethyleneimine for MRI contrast agent. They found that the *r*_2_ relaxivity of NRs is greater than nanoparticles using equal material volume. Iron oxides NWs outperform similar NPs while testing for *R*_2_ relaxitivities [5]. The calculated *R*_2_ value for NWs is 116 mM^−1^ s^−1^ which is higher than NPs (70 mM^−1^ s^−1^). Author concludes that the elongated nature and higher saturation magnetization of NWs result in improved MR contrast [5].

Despite of the fact that the general structural properties of core-shell iron nanostructures are well-studied, the exact structure and phase of oxide layers are hard to determine. The composition and phase of the oxide layer formed on an iron core depends on the distance from the inner Fe core to the outer oxide layers, i.e., usually a progression from zero valent Fe→FeO→Fe_3_O_4_→Fe_2_O_3_ occurs [6]. Although, for room temperature oxidation, a very thin oxide layer was formed which was hard to differentiate the spatial variation of oxide shells. For room temperature oxidation using EELS in TEM characterizations, some authors claim that the iron oxide shell is made of either *γ*-Fe_2_O_3_ or Fe_3_O_4_ [7, 8] or that FeO was initially formed and after heating the FeO shell was transformed into *γ*-Fe_2_O_3_ or Fe_3_O_4_ shell [9]. However *γ*-Fe_2_O_3_ and Fe_3_O_4_ are meta stable phases of iron and they could transform into *α*-Fe_2_O_3_ after high-temperature oxidation up to 500 °C [10]. Furthermore, the oxidation procedure of zero-valent iron core is strongly influenced by oxygenated aqueous solution and the oxidation occurs at an H_2_O and oxide interface [11]. As the transfer of iron ions occurs from the zero-valent iron core to the oxide shell, the formation of new oxide phase occurs. Similar oxidation was also found in [12] with the oxygen presence.

The purpose of this research was to study the structure of freshly prepared Fe NCs synthesized by the reduction of Iron(III) chloride solution by the addition of sodium borohydride solution. Study shows that the thickness of the shell and the diameter of the magnetic core of Fe NCs are tunable. XRD, FE-SEM, and TEM were used to study the structure and core-shell nature of Fe-NCs. Further, Raman spectroscopy with green laser (excitation wavelength 532 nm) and He-Ne laser (excitation wavelength 633 nm) was employed to study the oxide nature of Fe-NCs. The results were further confirmed by Mössbauer spectroscopy on FeNCs-0 and FeNCs-6 at 320 K. In order to study the magnetization effect on Fe-NCs, for future possible biomedical applications*,* the magnetic properties on Fe NCs-0, Fe NCs-2, Fe NCs-4, and Fe NCs-6 were measured by VSM at room temperature. After examining the saturation magnetization values of Fe NCs, it can possibly suggest that Fe NCs could be used as *r*_2_ contrast agents for magnetic resonance imaging (MRI) in the near future.

## Methods

### Chemicals

Ferric chloride hexahydrate (FeCl_3_.6H_2_O) (99% pure) and sodium boro-hydride (NaBH_4_) (98% pure) were purchased from National Medicines Corporation Ltd. (China). Highly pure argon gas (99.9%) was purchased from Hubei Minghui gas company (China).

### Synthesis of Core-Shell Fe NCs

For the synthesis of core-shell Fe NCs, 3 g of Ferric chloride hexahydrate (FeCl_3_.6H_2_O) was dissolved in 1000 ml of deionized (DI) water to form Iron(III) chloride solution. Sodium boro-hydride (NaBH_4_) solution was formed by adding 6 g of NaBH_4_ added to 400 mL of DI water [13]. The addition of NaBH_4_ solution was done at the rate of 1.5 mL/s in ferric solution without stirring and left the solution for 0 min, 120 min, 240 min, and 360 min. Black precipitates were formed, collected from the solution, and washed by DI water and ethanol and dried under argon gas (Ar) for characterizations. Fe NCs were formed by the reduction of Iron(III) chloride solution by adding sodium borohydride solution, which is done by following reaction [13],

$$ 3B{H}_4^{-}+3{H}_2O+ Fe{\left({H}_2O\right)}_6^3= Fe+3B{(OH)}_3+1/2{H}_2 $$

**Fig. 2:**
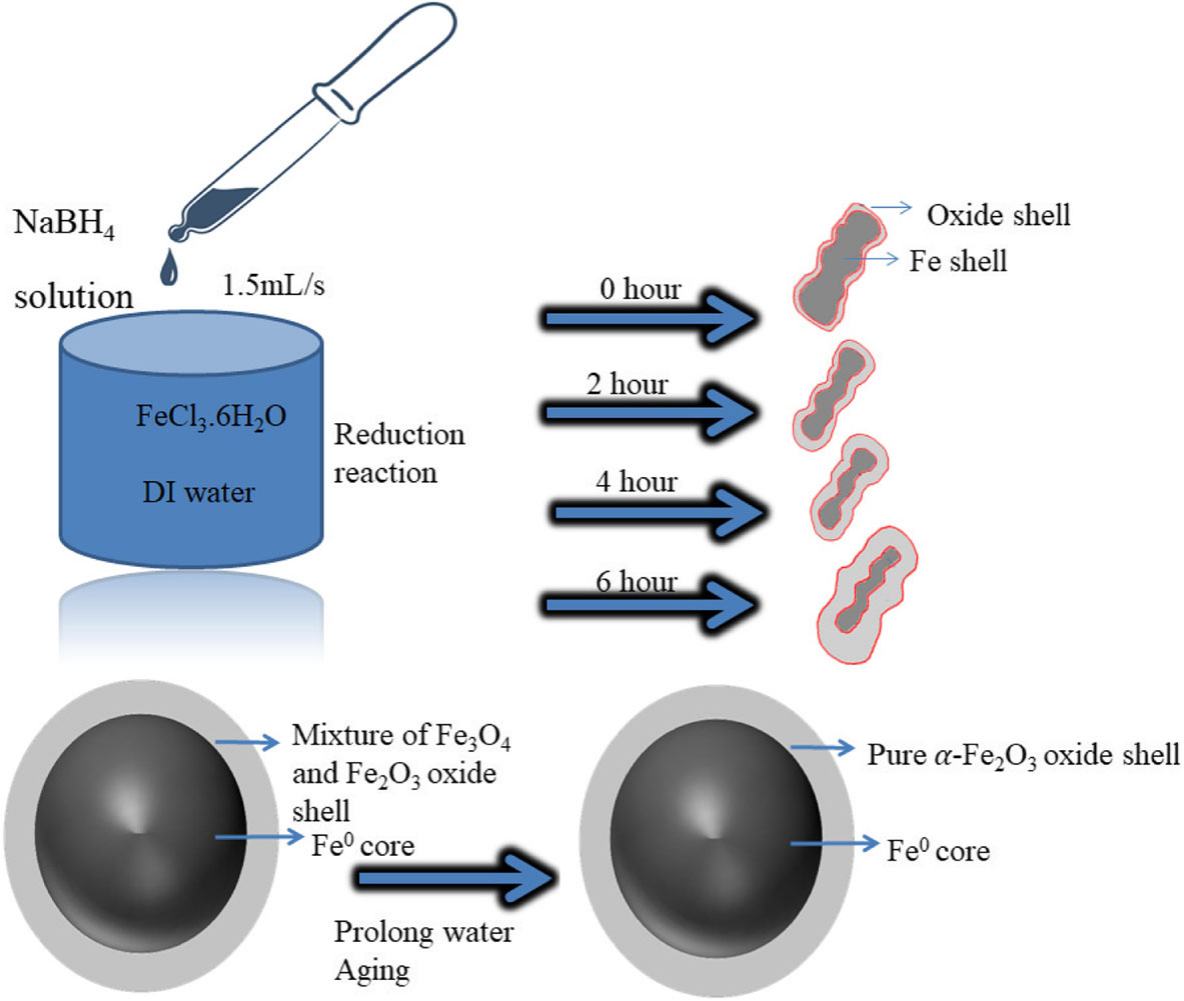
Schematic illustration of synthesis and formation of core-shell Fe NCs

By the addition of the NaBH_4_ solution in the ferric solution iron nuclei are formed under H_2_ protection. By self-assembling of iron nuclei because of high magnetic interaction between them, chain-like nanostructures were formed. Figure 2 shows the synthesis and formation of core-shell Fe NCs prepared through reduction reaction.

### Characterizations

The core-shell Fe NWs were examined by using field emission scanning electron miscroscopy (FE-SEM, NOVA 400 Nano) with energy-dispersive X-ray spectroscopy (EDX, Le350 PentaFETx–3). For FE-SEM observations the Fe NWs were sputtered with a thin layer of gold for 100 sec. X-ray diffraction (XRD, X’Pert PRO MRD, PANalytical, Netherlands) was done with Cu-K_α_ radiation, Raman spectrometer (lab RAM HR JY-Evaluation) with an excitation wavelength of 532 nm (with 60-mW power and 6-mW laser power) and with He-Ne laser (with 0.1, 0.6, 1 and 3-mW laser power). Transmission electron microscopy (TEM) was carried on a Tecnai G2 F30 S-TWIN electron microscope operating at 300 kV. For TEM observations, the samples were prepared by dispersing the powders in absolute ethanol by ultrasonic. Magnetic measurements were performed by using a vibrating sample magnetometer (VSM, Lake Shore 7307). The magnetization curves M/Ms Vs H(KOe) were measured under magnetic field up to 20 KOe. Mössbauer spectroscopy was performed by using 57Co/:Rhc ray source (14.4 KeV) mounted on an electromagnetic drive with triangular velocity signals at 320 K. The spectra were least squares fitted to get the hyperfine parameters.

## Results and Discussion

### FE-SEM

**Fig. 3:**
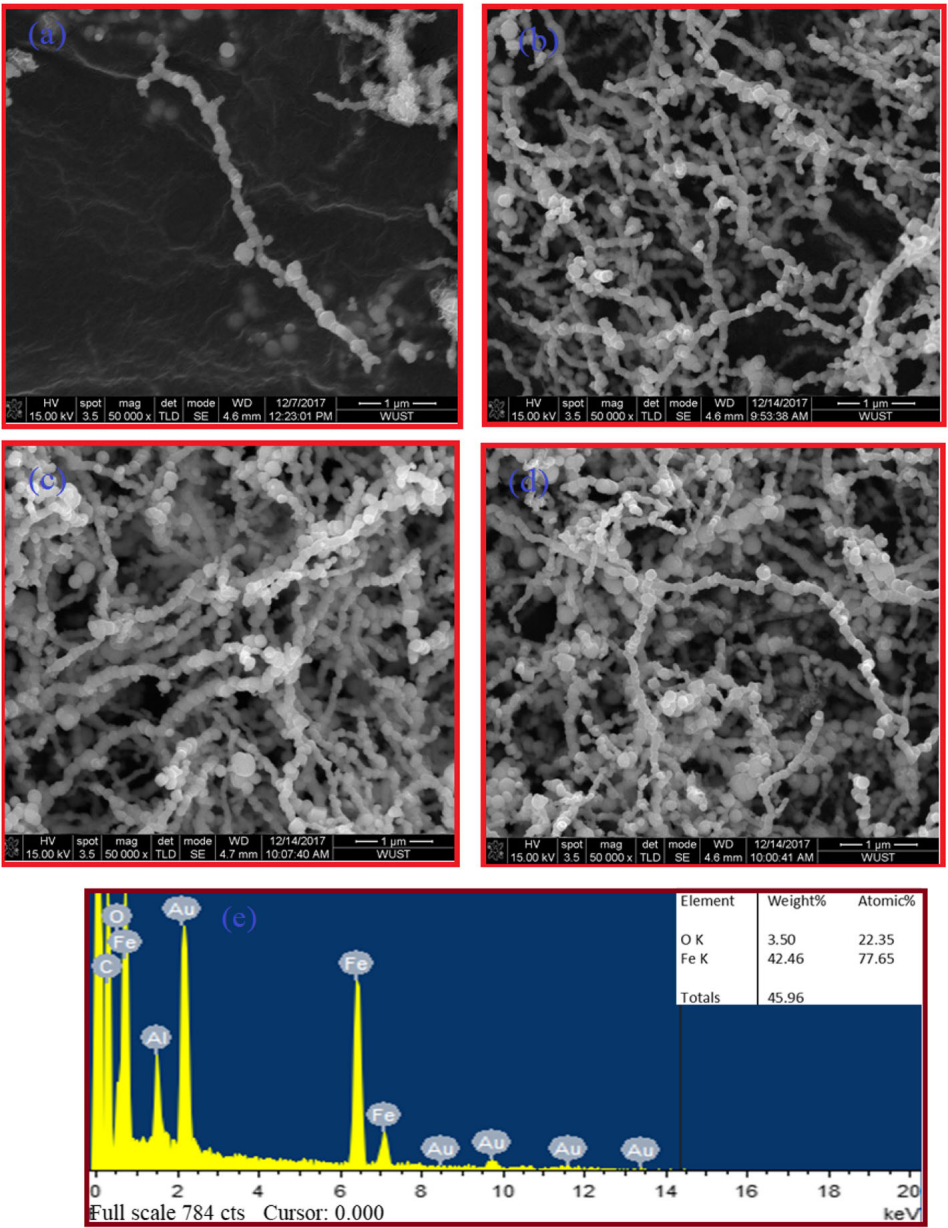
**a**–**e** FE-SEM image of (**a**) FeNCs–0, (**b**) FeNCs–2, (**c**) FeNCs–4, (**d**) FeNCs–6, (**e**) EDX pattern of Fe NCs–2, inset table show the atomic and weight percentage of iron and oxygen element

Figure 3a–d show an FESEM images of freshly prepared core-shell Fe NCs-0, Fe NCs-2, Fe NCs-4, and Fe NCs-6, through reduction reaction of iron tri-chloride by sodium borohydride at 0, 120, 240, and 360 min. One can see in Fig. 3a–d that the obtained Fe nanostructure appears like a chain of Fe nanoparticles linked together. Moreover, each of the Fe nanoparticles is separated from the other by a thin oxide interface, which is a specific feature of applied synthesis method used in this study. Figure 3e shows the composition of Fe NCs-2 measured using EDX. The iron (Fe) peak with oxygen (O) peak appears as shoulder to Fe peak which is observed, besides carbon (C) peak was observed due to carbon tape which was used as a substrate during FESEM characterization and gold (Au) peaks come from sputtering of sample with gold for conduction purpose. The inset of Fig. 3e shows the atomic percentage of Fe and O in Fe NCs-2 (At % = O, 22.35%; Fe, 77.65%).

### XRD

**Fig. 4:**
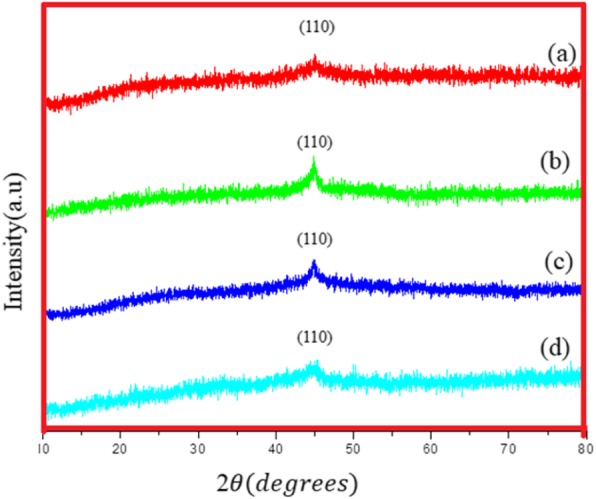
XRD pattern of (a) Fe NCs-0, (b) Fe NCs-2, (c) Fe NCs-4, and (d) Fe NCs-6

Figure 4a–d show X-ray patterns of Fe NCs-0, Fe NCs-2, Fe NCs-4, and Fe NCs-6.The dominant peak lies at 2*θ* = 44.9° can be unambiguously attributed to bcc Fe according to JCDP file no 6-696 with *a* = 0.2866A^°^. XRD is the most commonly used technique for the confirmation of the amorphous nature of iron oxide [14, 15]. A flat line shows the lack of periodicity in the crystal lattice. Therefore, the absence of Braggs diffraction peaks could be the identification marker of amorphous nature of Fe_2_O_3_ [15] and its distinction from polymorphs (hametite and maghemite) Fe_2_O_3_. In addition, it was seen from XRD figure that the characteristic peak of bcc Fe at 44.9° has a very slight shift towards lower angle with the increase of oxygen content in Fe NCs. This phenomenon could be observed due to the difference in ionic radii between the main element (Fe) and the dopant ion (O) [16], which indicates that O atoms have been successfully produced on the surface of Fe NCs. As the oxide peak does not appear in X-ray diffraction, the results were further confirmed by TEM, Raman spectroscopy, and Mössbauer spectroscopy.

### TEM

**Fig. 5:**
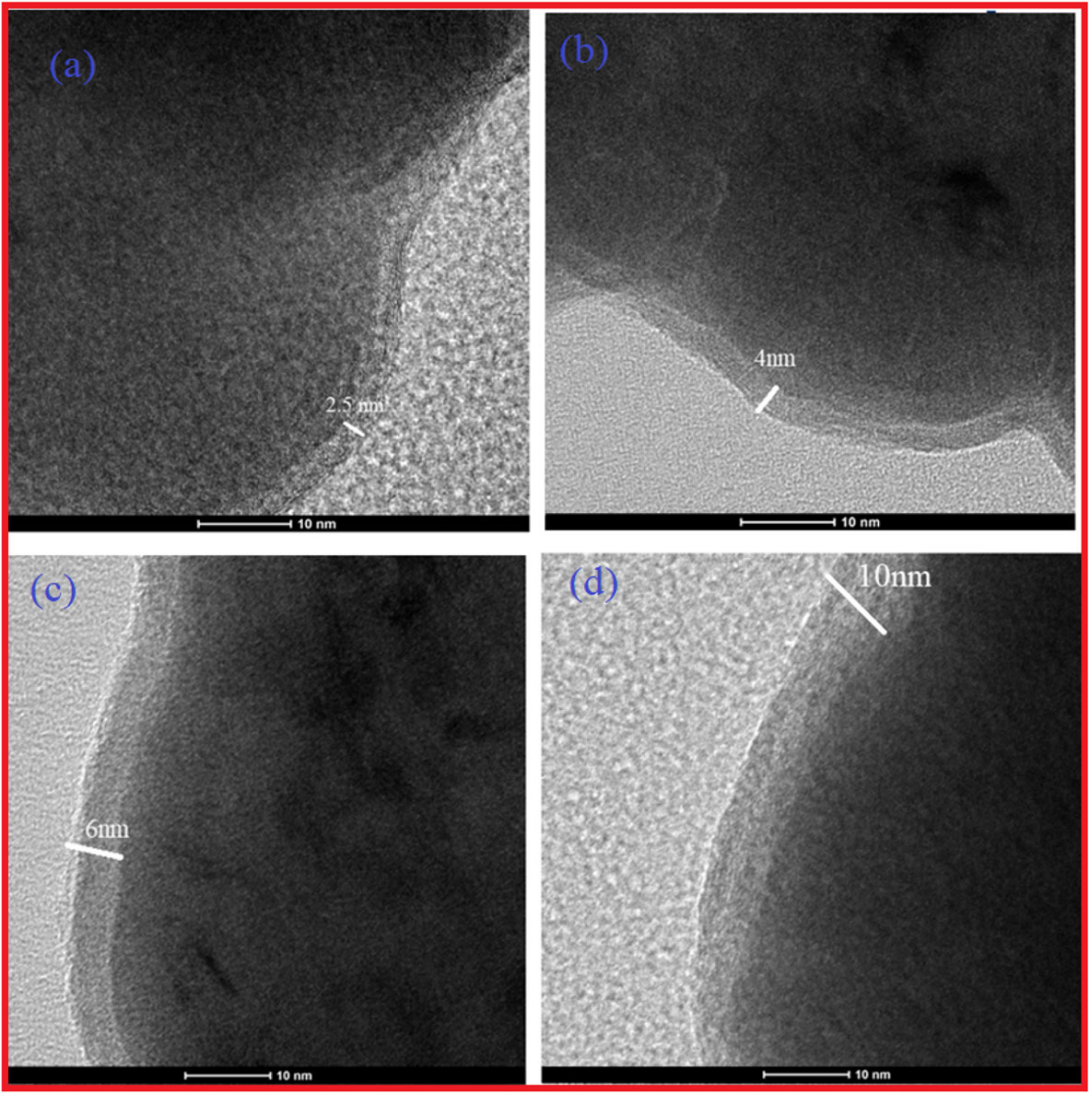
TEM image of **a** Fe NCs-0, **b** Fe NCs-2, **c** Fe NCs-4, and **d** Fe NCs-(6) showing core-shell nature of Fe NCs with an increase in oxide thickness of 2.5 nm, 4 nm, 6 nm and 10 nm

The result was further confirmed by TEM. Figure 5a–d shows the TEM of Fe NCs-0, Fe NCs-2,Fe NCs-4, Fe NCs-6. Remarkably, the contrast between dark center and gray edge was clearly seen, suggesting the core-shell structure of the NCs. As clear in Fig. 5a, the shell of Fe NCs-0 was about 2.5 nm, as the oxidation in water increases from 0 to 120 min, the shell increased in thickness to 4 nm (Fig. 5b), further increase in reaction time up to 240 min the oxide thickness of shell was increased to 6 nm (Fig. 5c), finally the aging in water for 360 min the oxide shell was increased up to 10 nm (Fig. 5 d). As clear from TEM observation, there is gradual increase in thickness of shell from 2.5, 4, 6 to 10 nm. It could be concluded that more water aging time results in a thicker oxide shell because of reaction of zero valent iron with O_2_/H_2_O in the solution. The increase in shell thickness was also seen by Xue et al. by preparing the core-shell nanocomposite, synthesized through the facile reflux method [17, 18].

### Raman Spectroscopy

**Fig. 6:**
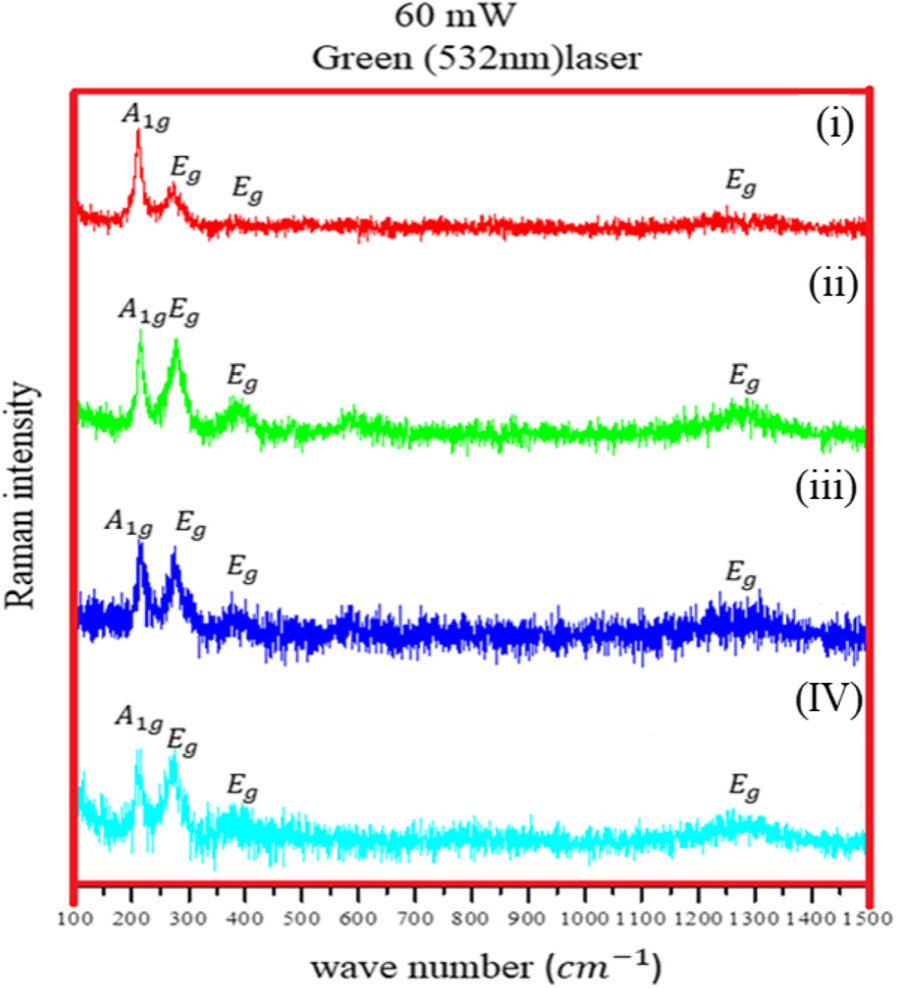
Raman Spectra of (i) FeNCs-0, (ii) FeNCs-2, (iii) FeNCs-4, (IV) FeNCs-6 at 60 mW laser power with Green laser

**Fig. 7:**
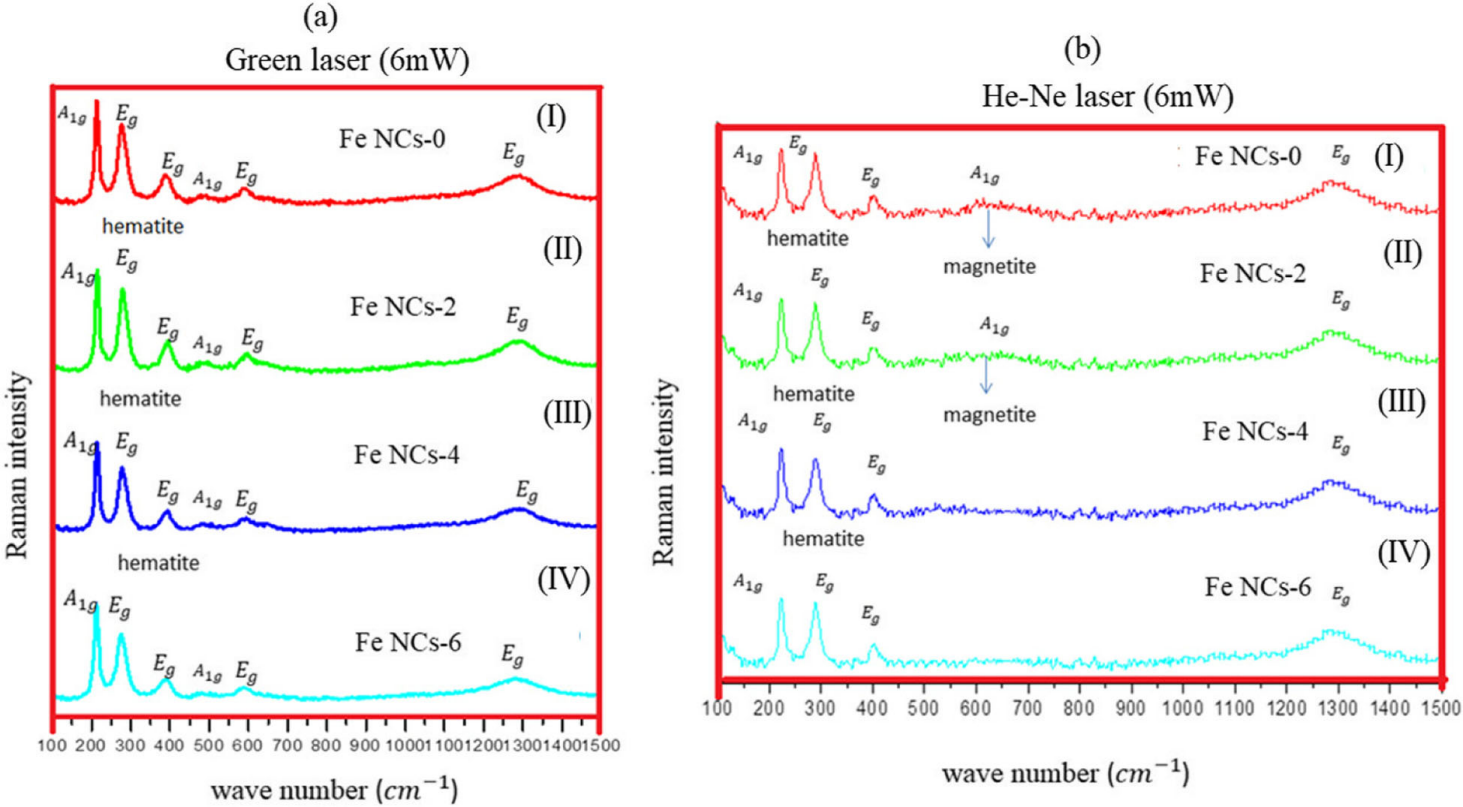
Raman Spectra of **a** Fe NCs-0 (I), Fe NCs-2 (II), Fe NCs-4 (III), and Fe NCs-6 (IV) collected at 6 mW laser power with Green laser and **b** Fe NCs-0 (I), Fe NCs-2 (II), Fe NCs-4 (III), and Fe NCs-6 (IV) collected at 6 mW laser power with He-Ne laser

**Fig. 8:**
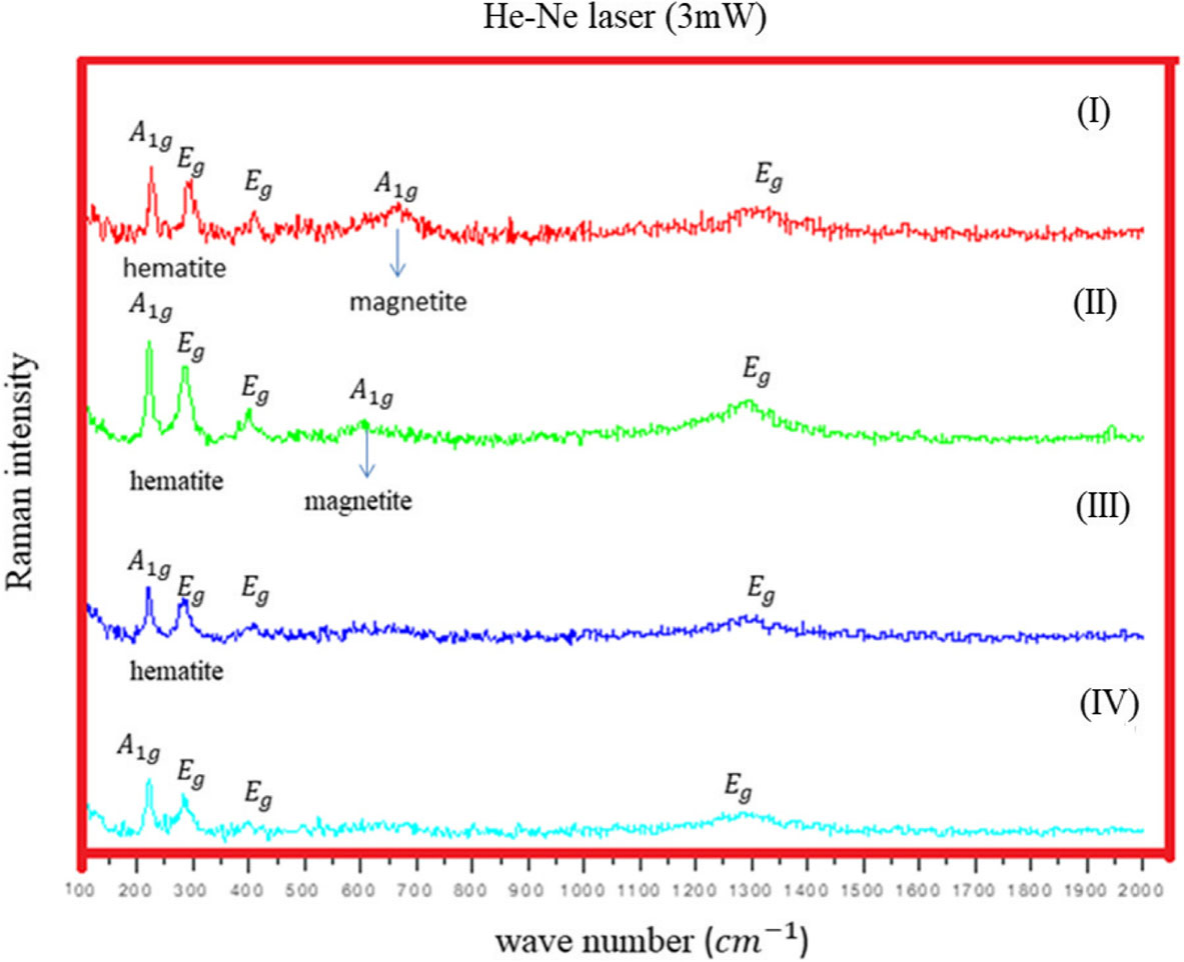
Raman Spectra of Fe NCs-0 (I), Fe NCs-2 (II), Fe NCs-4 (III), Fe NCs-6 (IV) at 3 mW laser power with He-Ne laser

Raman spectroscopy is an analytical technique which has been used to study the structure of iron oxides for many years [19–23]. It gives a clear assessment of the oxide phase and can measure crystallinity of the sample under study by observing the phonon modes. Raman vibrational spectroscopy is a great tool to characterize the oxide powders or films as previous studies show that polymorphs iron oxide (hematite, maghemite, and magnetite) shows distinct Raman signals [20, 24, 25]. Figure 6 shows collected Raman spectra from Fe NCs at 60-mW laser power, measured with green (532 nm) laser. Peaks appeared at 217 cm^−1^(A_1g_), 275 cm^−1^ (E_g_), and 386 cm^−1^ (E_g_), and a broad hump was observed between 1200 and 1300 cm^−1^, corresponds to hematite phase with peaks shifted towards lower wavenumbers (as the previous data collected in Table 1). This peak shift could correspond to laser oxidation; the high temperature induced by focused laser power could results in phase change of material which is also observed by group of studies by Mendili et al. [29–31]. To further confirm the oxide nature of Fe NCs, Raman spectra was done at lower (6 mW) laser powers using green laser (532 nm) and He-Ne (633 nm) laser. Figure 7 a shows (green laser) peaks at wavelength 214 cm^−1^(A_1g_), 278 cm^−1^ (E_g_), 394 cm^−1^ (E_g_), 490 cm^−1^ (A_1g_), 597 cm^−1^ (E_g_), and 1290 cm^−1^. As clear from Raman data collected in Table 1, these peaks could correspond to hematite peaks with wavenumber shifted towards the lower wavenumbers. So He-Ne laser (Fig. 7b) was used with 6-mW laser power. The peaks at wave number 224 cm^−1^ (A_1g_), 287 cm^−1^ (E_g_) and 484 cm^−1^ (A_1g_), and 1306 cm^−1^ corresponds to pure hematite phase [10, 24, 32]. By knowing the relation${P}_{\mathrm{scattered}}\propto \frac{I_0}{\lambda^4}$ (where *P*_scattered_ is Raman scattering time and *λ* is the laser wavelength), the scan time for He-Ne laser is longer than the green laser, which give better results for core-shell Fe NCs synthesized through reduction reaction. As clear in Fig. 7b, a weak peak appeared at 660 cm^−1^ for Fe NCs-0 and Fe NCs-2. This peak was seen by other groups in Raman spectra of hematite and it could be the presence of magnetite contamination [24, 33]. To further confirm the magnetite phase in Fe NCs the Raman spectra was collected with lower laser powers (0.1 mW, 0.6 mW,1 mW, and 2 mW) using He-Ne laser (results are given in Additional file 1). No Raman signals were seen, just a flat line was observed at low laser powers. The Raman spectra were collected at 3 mW using He-Ne laser as shown in Fig. 8. A strong magnetite peak clearly appears at 670 cm^−1^ (A_1g_) for Fe NCs-0 with hematite peaks at 224 cm^−1^ (A_1g_), 287 cm^−1^ (E_g_) 406 cm^−1^ (E_g_), 500 cm^−1^ (A_1g_), and 1310 cm^−1^ (E_g_) wavenumbers. Magnetite peak shifts towards lower wavenumber with lower intensities for Fe NCs-2 and disappears for Fe NCs-4 and Fe NCs-6. From Raman spectroscopy, it could be assumed that the shell of Fe NCs-4 and Fe NCs-6 correspond to pure hematite phase.

**Table 1:** Combined Raman data for iron oxides bands from [26–28]

Oxide phase	Wave number (cm^−1^)
αFe_2_O_3_ (hematite)	224(A_1g_), 249(E_g_), 287(E_g_), 406 (E_g_), 500 (A_1g_), 615 (E_g_), 660(LO Eu), 1310(A_1g_).
Fe_3_O_4_ (magnetite)	310(T_2g_), 538 (T_2g_), 668(A_1g_)
γFe_2_O_3_ (maghemite)	350(T_2g_), 500(E_g_), 700 (A_1g_)

### Mössbauer Spectra

**Fig. 9:**
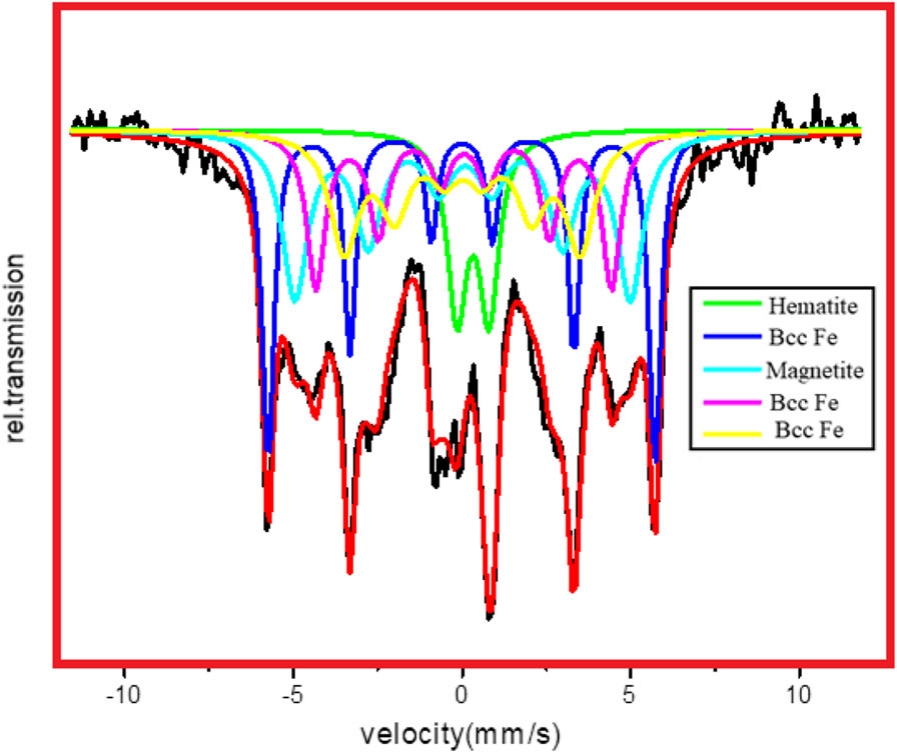
Mössbauer spectra of Fe NCs-0 at 320 K

**Fig. 10:**
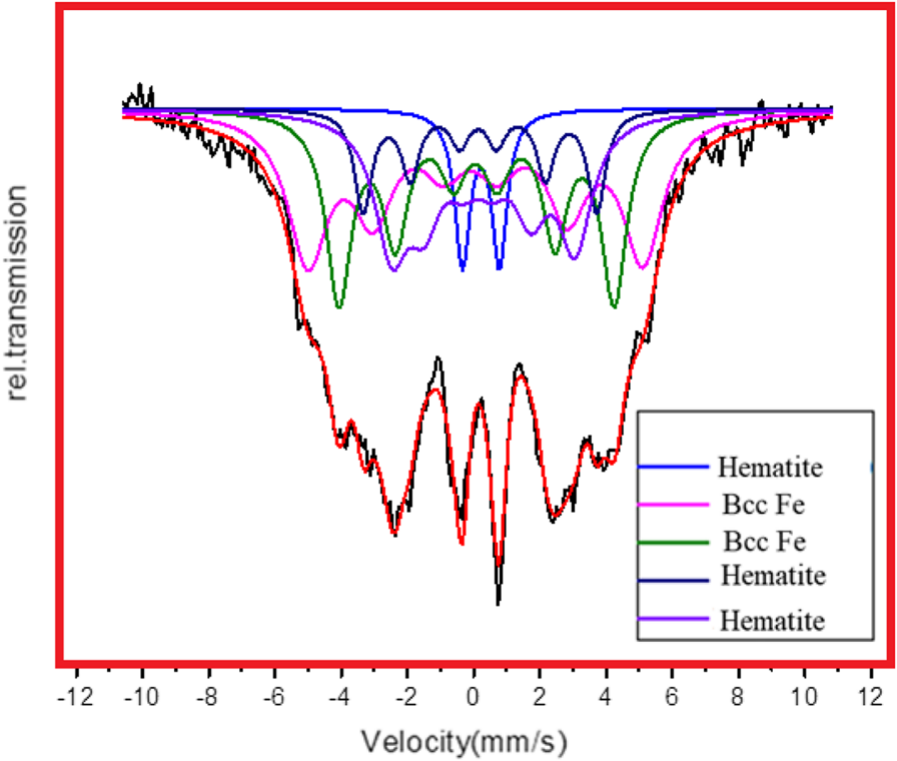
Mössbauer spectra of Fe NCs-6 at 320 K

In order to confirm the magnetite phase in Fe NCs, the Mössbauer spectroscopy was done on Fe NCs-0 and Fe NCs-6. The Mössbauer spectra for Fe NCs-0 are obtained at 320 K and are shown in Fig. 9. The Mössbauer spectra for Fe NCs-6 are obtained at 320 K and are shown in Fig. 10. Well-resolved six line spectra are observed in both spectra. The values of isomer shifts (*δ*), quadrupole splitting (*Q*), hyperfine field **(***H*), line widths and site population are deduced from the Mössbauer data. The best fits to the experimental data were obtained with one quadrupole doublet and four Zeeman sextets. The isomer shift gives the information about s electron charge distribution. A doublet with isomer shifts (*δ*) 0.24 mm/s and quadrupole splitting (*Q*) 0.94 mm/s, in Fe NCs-0, and in Fe NCs-6 with *δ* 0.21 mm/s and *Q* 1.11 mm/s ascribed to super paramagnetic Fe^+3^ state, also seen by other researchers while studying Fe_2_O_3_ nanoparticles [34–37]. The sextets specify magnetic spin states in Fe NCs. The *δ* values from 0.7 to 1.4 mm/s are ascribed to Fe^+2^ and values from 0.1 to 0.6 mm/s indicating iron in Fe^+3^ state [38–41]. *δ* values given in Table 2 for sextet 2 is 0.10 mm/s and for Q is − 0.04 mm/s which shows valence state of iron is +3. Similar values are also observed by Joos et al. [42] for Fe_3_O_4_ nanoparticles; they ascribed these values to tetrahedral Fe^+3^. *δ* values for Fe NCs-6 given in Table 3 for sextet 3 and 4 are 0.15 mm/s and 0.20 mm/s shows Fe^+3^ in Fe_2_O_3_. Xie et al. [43] ascribed similar values for larger Fe_2_O_3_ nanoparticles. It could be deduced from the Mössbauer data that there is small contamination of magnetite which is present in Fe NCs-0 and Fe NCs-6 and corresponds to pure Hematite shell. Korecki and Gradmann [44] did Mössbauer spectroscopy on Fe(110) films, obtained isomer shift values are 0.02 mm/s, 0.04 mm/s, and 0.07 mm/s. These values matched well with bcc Fe values given in Table 2 and Table 3 for Fe NCs-0 and Fe NCs-6. This is expected that the Fe concentration in the Fe NCs will decrease as the oxide shell increases from 2.5 to 10 nm. The site population of Fe decreases and site population of hematite increases significantly in Fe NCs-6. The quadrupole splitting and line widths also change with the increase in shell thickness of Fe NCs-0 to Fe NCs-6.

**Table 2:** Mössbauer parameters, isomer shifts (*δ*), quadrupole splitting (*Q*), and hyperfine field (*H*) of Fe NCs-0.

	*δ* (mm/s)	*Q* (mm/s)	*H* (kOe)	Line width (mm/s)	Site population	Possible assessment
Doublet	0.249	0.945		0.167	1.77	Hematite
Sextet-1	0.032	− 0.022	326	0.286	9.0	Bcc Fe
Sextet-2	0.101	− 0.04	278.3	0.326	11.9	Magnetite
Sextet-3	0.062	− 0.012	238.4	0.33	11.5	Bcc Fe
Sextet-4	0.070	− 0.019	207	2.03	65.9	Bcc Fe

**Table 3:** Mössbauer parameters, isomer shifts (*δ*), quadrupole splitting (*Q*), and hyperfine field (*H*) of Fe NCs-6

	*δ* (mm/s)	*Q* (mm/s)	*H* (kOe)	Line width (mm/s)	Site population	Possible assessment
Doublet	0.218	1.119	–	0.273	6.44	Hematite
Sextet-1	− 0.028	0.077	313.9	0.73	32.9	Bcc Fe
Sextet-2	0.063	0.020	257.8	0.461	26.6	Bcc Fe
Sextet-3	0.158	0.030	218.9	0.32	9.9	Hematite
Sextet-4	0.202	0.089	172	0.61	24.1	Hematite

**Scheme 1:**
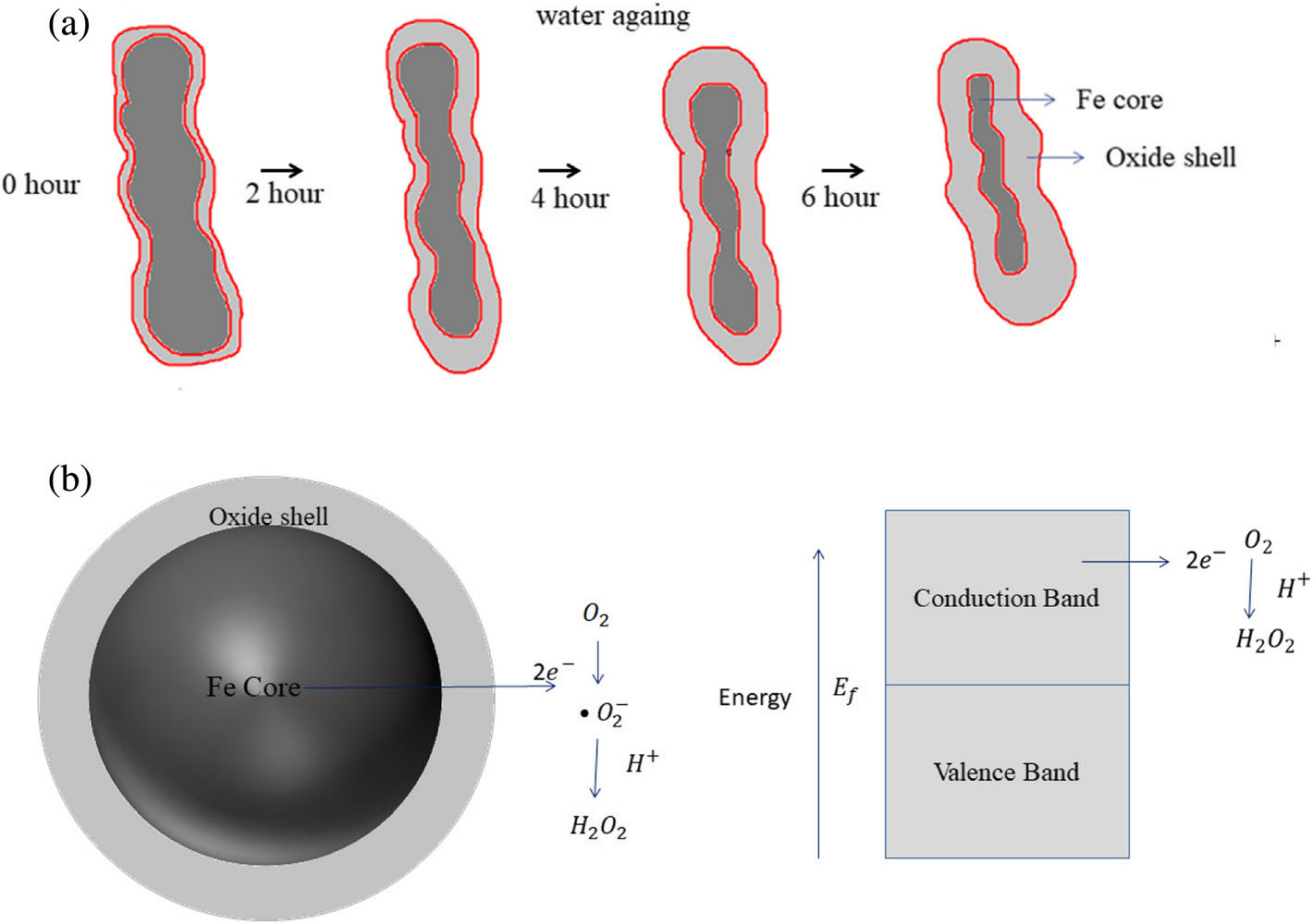
**a** Formation of Fe NCs prepared through 120, 240 and 360 min of water aging, **b** mechanism of O_2_ activation route over the core-shell Fe NCs

The schematic illustration (Scheme 1) depicts the mechanism for the formation of core-shell Fe NCs explained by O_2_ activation route by oxidation of Fe NCs in water. Scheme 1a shows that when zero valent Fe were exposed to H_2_O and O_2_ the iron oxide layer was produced on the surface of Fe NC. As the reaction time in water increases from 0 to 360 min, the growth of the oxide layer increases and the Fe core decreases. The oxidation of the Fe core happens due the transfer of electrons from the Fe core to the iron oxide shell through conduction band. The electron transfer occurs due to the work function effect. As the work function of Fe (4.5 eV) is lower than magnetite (5.52 eV) and hematite (5.6 eV), the electron transfer occurs in order to lower the energies so the position of Fermi level was adjusted [45, 46]. The oxidation of zero valent Fe to Fe^+2^ ions (ferrous ions) was followed with the transfer of 2 electrons from core of Fe NCs to oxygen gas in the solution to produce hydrogen peroxide (Scheme 1b) [47].

$$ {Fe}^0+{O}_2+2{H}^{+}\to {Fe}^{2+}+{H}_2{O}_2 $$

Further, H_2_O_2_ reacts with ferrous ions to produce hydroxyl radical and ferric ions [47].

$$ {Fe}^{2+}+{H}_2{O}_2\to {Fe}^{3+}+{OH}^{-}+\bullet \kern0.5em OH $$

The electron transfer rate could be affected by increase of the thickness of the oxide shell. The TEM analysis show that as the reaction time in the water increases, the thickness of oxide shell increases which could stops the electron transfer rate further. At the temperature below 150 °C, the electron transfer occurs mostly by electron tunneling results in formation of the oxide layer up to a few nanometers [48]. So after prolonged water oxidation, a stable oxide layer was formed on the surface of Fe core, because during the synthesis, the obtained precipitates of Fe NCs was dried under the inert atmosphere (in our case, Argon) to reduce the risk of further oxidation. These Fe NCs can be stable up to 6 months without further oxidation, which make them biocompatible and suitable candidates for biomedical applications.

### VSM

**Fig. 11:**
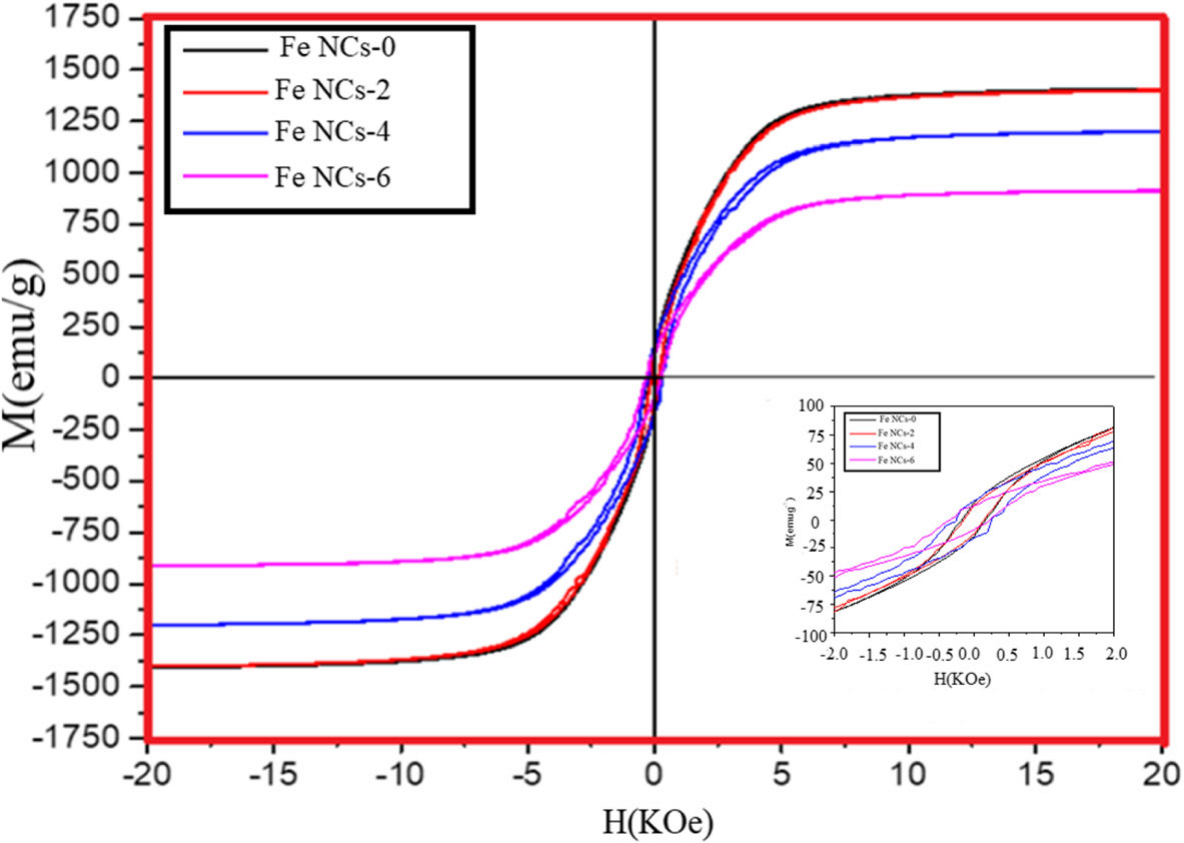
Magnetic hysteresis measured at room temperature, inset shows the enlarged curve between – 2.0 and 2.0 kOe

**Fig. 12:**
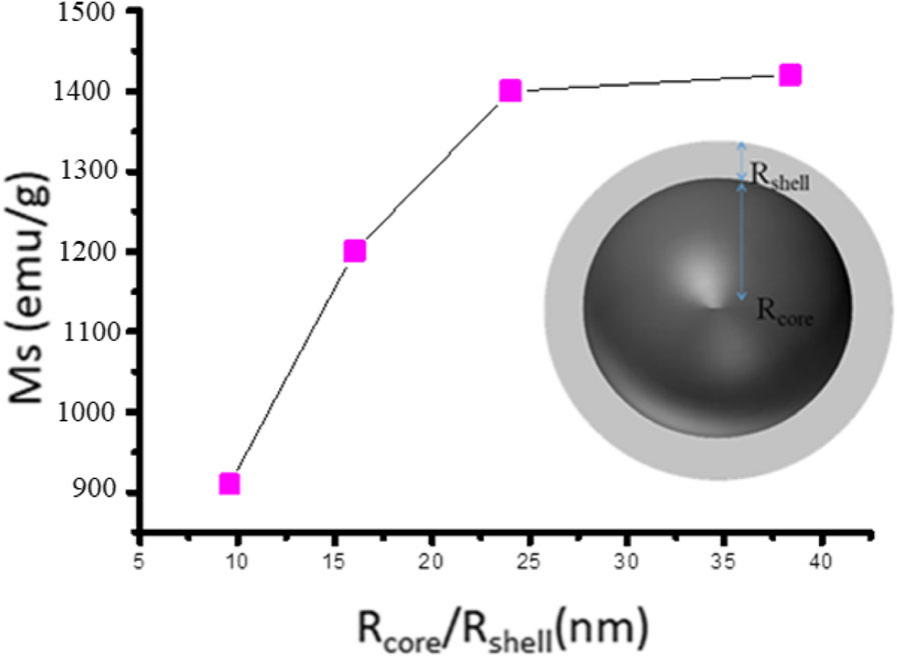
Ms Vs R_core_ /R_shell_ curve, taking the average diameter of Fe NCs 96 nm

The Magnetic properties of the Fe NCs were measured at 320 K as shown in Fig. 11. It is clear from the Fig. 11 that the Fe NCs-0 and Fe NCs-2 possess the saturation magnetizing (Ms) value of 1400 emu/g and 1420 emu/g, which is higher than Fe NCs-4 and Fe NCs-6 with Ms values of 1200 emu/g and 910 emu/g. In the initial stage of oxidation (for 0 min and 120 min), the shell of Fe NCs-0 and Fe NCs-2 was made of a mixture of magnetite and hematite which cause reduction in contribution of zero valent Fe core, but due to presence of magnetite in both samples they possess high magnetic moment, so the saturation magnetization value of Fe NCs-0 is nearer to Fe NCs-2. But further increase in water aging (from 120, 240 to 360 min), the magnetite is gradually converted in to hematite_,_ and also the core of Fe decreases, so the saturation magnetization of samples Fe NCs-2, FeNCs-4, and Fe NCs-6 decreases as well (i.e., 1420 emu/, 1200 emu/g, 910 emu/g). Figure 12 shows the dependence of the Ms (emu/g) values on increasing the thickness of the oxidized shell for Fe NCs-0, Fe NCs-2, Fe NCs-4, and Fe NCs-6 having an average similar diameter of 96 nm and different R_core_/R_shell_ ratio (Table 4).

**Table 4:** Measured values of saturation magnetization (Ms), Coercivity (Hc), and Remanent magnetization (Mr) from the Hysteresis loop of Fig. 14

Fe-NCs	Mr (emu/g)	Hc (Oe)	Ms (emu/g)
Fe NCs-0	0.152	158	1420
Fe NCs-2	0.143	147.22	1400
Fe NCs-4	0.106	179.54	1200
Fe NCs-6	0.109	194.42	910

For explaining the magnetization reversal phenomena in chain composed of single domain spherical particles, Jacobs and Beans [49] introduced the “Chain of spheres model.” The chain of spheres model could apply for Fe NCs, by considering the chain-like assembly of Fe nanoparticles [49]. Though the module is only applicable to the single domain particles with long-range dipolar interactions between, in this case, the Hc values for Fe NCs are considered very low compared to the values predicted in [49]. The two important mechanisms, fanning mechanism, and curling mechanism are related two magnetization eigenstate. In our case, both of the mechanisms are not applicable. Recently Krajewski et al. [50] have studied structural and magnetic properties of Fe NCs and NPs and predicted that Fe NCs follow the fanning mechanism. Though (1) unlike Zhang and Manthirama’s [51] results, their study did not provide the proof that Fe NCs lie in the single domain region (i.e., Zhang and Manthirama results show that the Hc values of Fe NCs increases with increase of particle diameter, and in the diameter range of 28–35 nm, the Hc value stays almost maximum (≈ 1250 Oe), this is the region which corresponds to a single domain of Fe NCs). (2) Secondly, the Hc value in their study [50] found very low (i.e., 300 Oe) than previous theoretical studies explaining the fanning mechanism (i.e., Hc ≥ 900 Oe for *n* = 2–∞, where *n* is the number of particles in the chain) [49]. (3) Finally, there is no explanation on magnetization reversal behavior of Fe NCs.

Previous work show that for MRI applications nanoparticles morphology (Ms value, size, and dopant material) was strongly considered for enhancing the *r*_2_ values [52–54]. The quantum mechanical outer sphere theory explains that the *r*_2_ relaxivity depends on both the effective radius and the Ms value of superparamagnetic core [55, 56]. The relaxivity *r*_2_ of superparamagnetic Fe NPs can be explained by [57–60]

1 $$ {r}_2=\frac{\left(256{\pi}^2{\gamma}^2\right){kM}_s^2{r}^2}{D\left(1+\frac{L}{r}\right)} $$

where *γ* and Ms are saturation magnetization, *r* is the radius of the magnetic core, *L* is the thickness, *D* is the diffusivity of water molecules, and *k* is the conversion factor. This equation shows that *r*_2_ values directly depend on Ms values and radius of magnetic material. Keeping this in view, Fe NCs-0 could be a potential candidate for *r*_2_ contrast agents for MRI in the near future.

## Conclusion

The structural analysis of Fe NCs was done by XRD, TEM, Raman spectroscopy, and Mössbauer spectroscopy. XRD analysis show that the core of Fe NCs was made by bcc Fe, but no iron oxide peak was observed. TEM results show a thin oxide layer was formed on Fe NCs, confirming the core-shell nature of Fe NCs. With the increase of reaction time in water from 0 to 360 min the oxide layer thickness increases from 2.5 to 10 nm. Raman studies show that shell of Fe NCs-0 and Fe NCs-2 was mixture of hematite and magnetite phase. The magnetite peak seems to disappear for Fe NCs-4 and Fe NCs-6. By analyzing the Mössbauer spectroscopy on Fe NCs-0 and Fe NCs-6, it was observed that the core of Fe NCs-0 and Fe NCs-6 was made of bcc Fe. The shell of Fe NCs-0 was made of magnetite, and hematite phase and shell of Fe NCs-6 show pure hematite phase. The possible mechanism for the formation of core-shell Fe NCs as deduced from Mössbauer spectroscopy and Raman spectroscopy is initial time of oxidation; the zero valent Fe core was immediately covered by a layer of magnetite and hematite shell, but due to prolonged water oxidation time the magnetite was gradually converted in to hematite. The magnetic properties of the Fe NCs were measured by VSM at room temperature. Ms values decrease with the increase of oxide shell, due to an increase in contribution of less magnetic moment hematite phase.

## Additional File

Additional file 1: Raman spectra was collected with lower laser powers (0.1mW, 0.6mW, 1mW and 2mW) using He-Ne laser. (DOCX 47 kb)

## Data Availability

The datasets supporting the conclusions of this article are included within the article.

## Abbreviations

0-D: Zero dimensional
1-D: One dimensional
DI: Deionized
EDX: Energy-dispersive X-ray spectroscopy
FE-SEM: Field emission scanning electron miscroscopy
H: Hyperfine field
Hc: Coercivity
MH: Magnetic hyperthermia
Mr: Remanent magnetization
MRI: Magnetic resonance imaging
Ms: Saturation magnetization
NCs: Nanochains
NWs: Nanowires
Q: Quadrupole splitting
TEM: Transmission electron microscopy
VSM: Vibrating sample magnetometer
XRD: X-ray diffraction
*δ*: Isomer shifts
