# Supplementary material for: Structural characterization of self-assembled chain like Fe-FeOx Core shell nanostructure
Source: Nanoscale Res Lett. 2019 Sep 9;14:308. doi: 10.1186/s11671-019-3128-2 (PMC6734011; doi:10.1186/s11671-019-3128-2)

Supplementary material

Raman spectra was collected with lower laser powers (0.1mW, 0.6mW, 1mW and 2mW) using He-Ne laser.


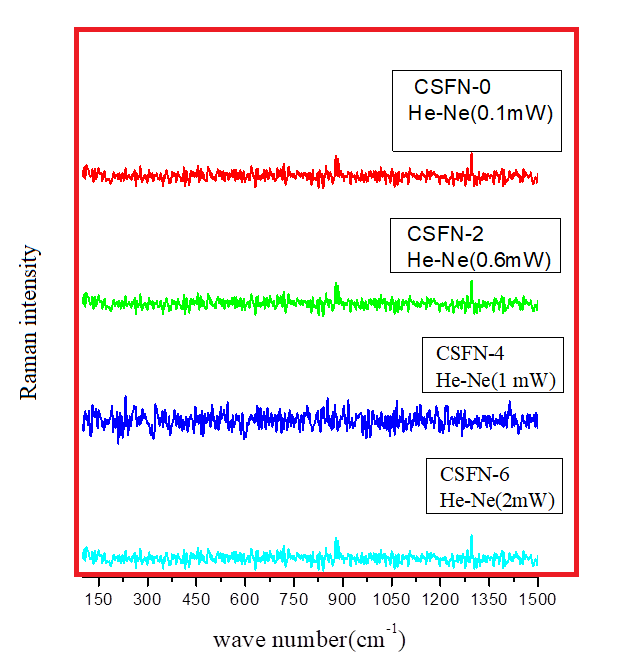

Supplement: Supplementary file 1 — Raman spectra was collected with lower laser powers (0.1mW, 0.6mW, 1mW and 2mW) using He-Ne laser. (DOCX 47 kb) [file 11671_2019_3128_MOESM1_ESM.docx]
